# Volumetric Comparison of the Temporomandibular Joint Space in Skeletal Class I and II Patients Using Cone-Beam Computed Tomography: A Cross-Sectional Study

**DOI:** 10.7759/cureus.75597

**Published:** 2024-12-12

**Authors:** Kanchan Das, Jasleen Kour, Bhanu Priya, Sudhanshu Mishra, Pritismita Das, Shalini Priya, Seema Gupta

**Affiliations:** 1 Department of Orthodontics, Tripura Medical College and Dr. B.R. Ambedkar Memorial (BRAM) Teaching Hospital, Agartala, IND; 2 Department of Dentistry, Shri Mata Vaishno Devi Institute of Medical Excellence, Kakrayal, IND; 3 Department of Oral Medicine and Radiology, Manipal College of Dental Sciences, Manipal, IND; 4 Department of Dentistry, Shobha Dental Care, Tanda, IND; 5 Department of Orthodontics, Kothiwal Dental College and Research Centre, Moradabad, IND

**Keywords:** cone-beam computed tomography (cbct), malocclusion, skeletal, temporomandibular joint, volumetric

## Abstract

Introduction: The temporomandibular joint (TMJ) is one of the most intricate anatomical entities within the human body and is clinically relevant in the field of dentistry. Therefore, the present study aimed to conduct a three-dimensional (3D) volumetric comparison of the TMJ space in skeletal Class I and II patients using cone-beam computed tomography (CBCT).

Materials and methods: This cross-sectional, retrospective study was conducted on 40 CBCT records divided into skeletal Class I and skeletal Class II patients. Volumetric assessment of the anterior, posterior, medial, and lateral joint spaces and condylar shape was performed. The study groups, consisting of Class I and Class II malocclusion patients, were compared using a two-way analysis of independent t-tests. Statistical significance was determined at a threshold of p < 0.05. Additionally, regression analysis was conducted to identify the variable that most strongly influenced the volume of TMJ space.

Results: Skeletal Class I patients showed statistically significant greater TMJ space volume of 1621.45±138.06 mm³, compared to 1483.55±138.29 mm³in skeletal Class II patients. The volume of the anterior and medial TMJ space was significantly larger in Class I of 878.35 ± 80.61 mm³and 855.40 ± 76.63 mm³,respectively, in contrast to Class II of 614.65 ± 65.81 mm³ and 532.85 ± 73.16 mm³. An oval condylar shape was associated with an increased total space volume. Age and gender did not show any significant correlation with TMJ volume. Multivariate linear regression analysis indicated significant correlations between malocclusion and the TMJ space volume.

Conclusion: The results of this study indicated that the condyle was positioned in an anterior and medial orientation in Class II patients when juxtaposed with Class I patients. Oval configuration was the most frequently observed morphology of the mandibular condyle.

## Introduction

The temporomandibular joint (TMJ) is integral to mandibular functionality, facilitating intricate movements that are vital for mastication, phonation, and general oral well-being. Among the frequently observed occlusal anomalies are skeletal class I and II malocclusions, each presenting unique attributes that affect the morphology and functionality of the TMJ [1]. Although conventional imaging techniques have yielded a significant understanding of the anatomy of the TMJ, recent innovations in cone-beam computed tomography (CBCT) and three-dimensional (3D) volumetric assessments have transformed our capacity to visualize and scrutinize TMJ structures with an unparalleled level of detail [2].

Skeletal Class II malocclusion is characterized by a retrusive mandible, often leading to altered TMJ loading patterns and potential morphological adaptations or pathologies [3]. Conversely, skeletal Class I malocclusion presents with relatively normal sagittal jaw relationships but may still exhibit TMJ irregularities owing to functional or positional abnormalities [4]. Understanding how these distinct skeletal classifications influence TMJ anatomy is essential for diagnosis, treatment planning, and long-term management of malocclusion-related disorders.

Cone-beam computed tomography technology mitigates the constraints inherent in traditional two-dimensional (2D) radiography by offering high-resolution, 3D visualization of craniofacial anatomy while simultaneously minimizing radiation exposure when compared to standard CT scans [5]. This advancement permits healthcare professionals and researchers to scrutinize the components of the TMJ, such as the mandibular condyle, articular disc, and glenoid fossa, with significantly improved precision [2]. Volumetric assessment enhances the applicability of CBCT by providing quantitative measurements of TMJ structures, thereby yielding objective information regarding condylar volume, surface area, and morphology along with the dimensions of the joint space [6]. This methodology is crucial for discerning subtle anatomical variations between skeletal Class I and Class II patients, which may not be evident through subjective assessments.

The application of 3D volumetric analysis in the examination of the TMJ is of considerable importance in the disciplines of orthodontics, prosthodontics, and oral surgical interventions. By employing CBCT in conjunction with 3D volumetric assessment, clinicians are afforded the opportunity to achieve a deeper understanding of the TMJ anatomy, thus enhancing the accuracy of diagnostic evaluations and treatment outcomes. Therefore, this study aimed to compare TMJ morphology in skeletal Class I and Class II patients using CBCT-based 3D volumetric analysis to enhance our understanding of TMJ adaptations in malocclusion and to provide evidence-based insights for optimizing clinical interventions.

## Materials and methods

Study design and setting

This retrospective study was conducted using CBCT records of skeletal Class II patients with retrognathic mandibles who visited the Department of Orthodontics between July 2018 and May 2024. This study was approved by the Institutional Ethics Review Board (IERB) of the Kothiwal Dental College and Research Centre, Moradabad, India (approval number: KDCRC/IERB/06/2024/56), and followed the principles of the Declaration of Helsinki. Written informed consent was obtained from all patients as a routine procedure to use their records for research purposes while maintaining confidentiality.

Eligibility criteria

Those with complete records having dentoskeletal Class I (angle between points A and B as the deepest point in the anterior concavity of maxilla and mandible and nasion as the most anterior point on frontonasal suture as ANB of 2°-4°), skeletal Class II patients with an average growth pattern (ANB > 4°, mandibular plane angle as SN-GoGn < 27°), age > 20 years, irrespective of sex, absence of any TMJ abnormality, and no degenerative diseases of the condyle, were included in the present study. Patients with incomplete records, a history of previous orthodontic treatment, TMJ trauma, mandibular asymmetry, skeletal Class III, congenital abnormalities such as cleft lip and palate or other craniofacial anomalies, and those requiring orthodontic camouflage were excluded from the study.

Sample size estimation

The sample size was calculated using G*Power software version 3.2.9 (Heinrich-Heine-Universität Düsseldorf, Düsseldorf, Germany). Based on an effect size of 0.80, derived from cephalometric SNA angle measurement comparison between Class I and Class II patients reported in a previous study [7], a minimum of 40 samples (20 in each group) was determined to be adequate.

Methodology

The CBCT scans were conducted following standard protocol, using a Carestream New Generation CBCT apparatus (Carestream Dental, Atlanta, GA, USA) in accordance with a standardized protocol (operating at a voltage of 120 kV, a current of 80 mA, a seven-second scan time, a field of view (FOV) measuring 10 × 10 cm, a resolution of 0.2 voxels, and 1-mm slice thickness). To achieve consistent head orientation, the subjects were positioned supine with the application of a head stabilizer. During CBCT scanning, patients were given specific instructions to maintain an erect seated posture with the teeth on maximum intercuspation and to not swallow. The eyelids and nasal dorsum were employed as the horizontal and vertical reference axes, respectively, and were established using a laser beam. Image reconstruction for visual analysis was performed using the CS Imaging Software, version 8 (Carestream Dental). All the CBCT scan records were utilized with the same parameters.

Three-dimensional volumetric analysis of joint space

Slicing was performed using the Carestream software. The CBCT axial plane Digital Imaging and Communications in Medicine (DICOM) files, with a resolution of 429 × 429 pixels at a slice thickness of 300 µm, were imported into the ImageJ software, version 1.54f (Wayne Rasband, NIH, Bethesda, MD, USA) for analysis. For consistent orientation, all three axes (axial, sagittal, and coronal) were aligned to pass through the center of the TMJ condyle. The landmarks and planes used in this study are listed in Table 1 and were obtained from previous studies [8, 9]. 

**Table 1:** Reference points and planes used in the study References: [8,9]

Reference points and planes	Definition of points and planes
Nasion (N)	Intersection point of frontal bone and nasal bone
Point A	Deepest point in anterior concavity of maxilla
Point B	Deepest point in anterior concavity of mandible
Point S	Mid-point of sella turcica
Point Go	Constructed point at intersection of ramal and mandibular plane
Point Gn	Anteroinferior point on bony symphysis
Orbitale (Or)	The most inferior point of the bony orbitale (Right: OrR, Left: OrL)
Porion (Po)	The most superior point of the external auditory meatus (Right: PoR, Left: PoL)
Basion (Ba)	Midpoint of the anterior margin of the foramen magnum on the occipital bone
Crista galli (Cg)	The most superior point of the crista galli located in the ethmoid bone
Lateral lip of eminence (Em)	The most inferior and lateral point of articular eminence (Right: EmR, Left: EmL)
Glenoid fossa (Gf)	The most superior point of the glenoid fossa (Right: GfR, Left: GfL)
Mid-axial plane (MAP)	A plane passing through OrR, OrL, and PoR
Mid-sagittal plane (MSP)	A plane perpendicular to MAP with passing through Cg and Ba
Coronal plane (COP)	A plane perpendicular to MAP and MSP with passing through Ba
ANB angle	Angle between point N to A, and N to B
SN-GoGn angle	Mandibular plane angle as angle between SN plane and line joining points Go and Gn
Eminence plane (EmP)	A plane parallel to MAP with passing through Em (Right: EmPR, Left: EmPL)
Glenoid fossa sagittal plane (GfSP)	A plane parallel to MSP with passing through Gf (Right: GfSPR, Left: GfSPL)
Glenoid fossa coronal plane (GfCP)	A plane parallel to COP with passing through Gf (Right: GfCPR, Left: GfCPL)

**Figure 1:**
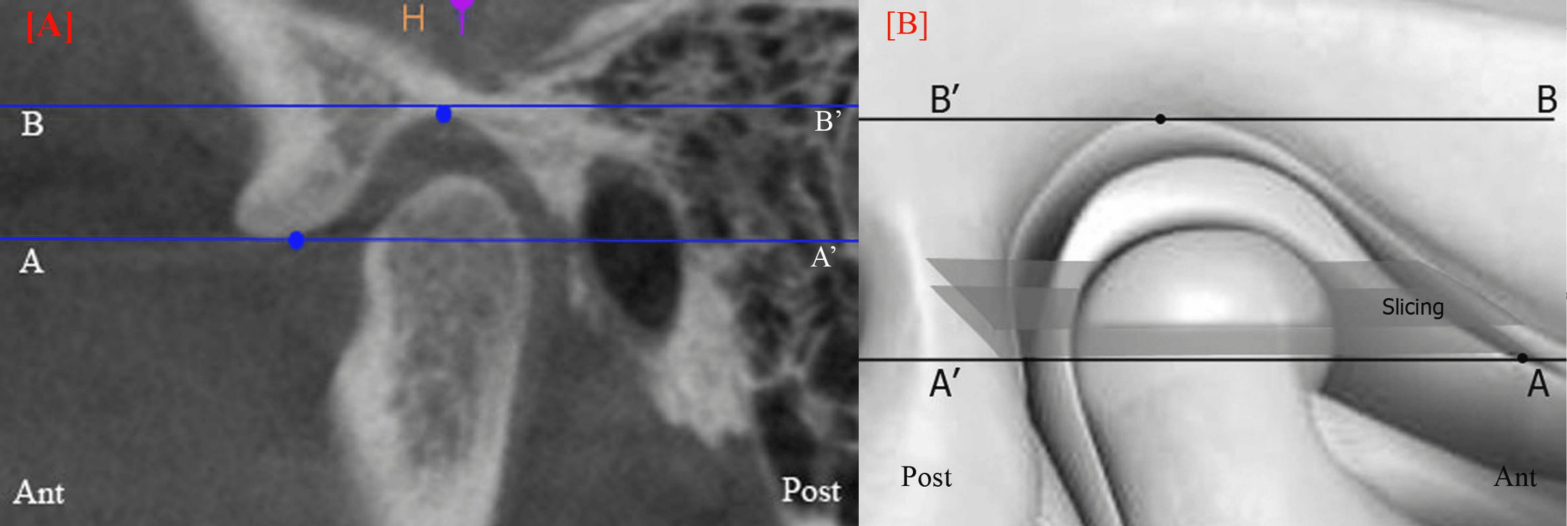
Preparation of slices for TMJ volumetric measurement A. TMJ thickness between two parallel lines (AA’ and BB’) was sliced at a slice thickness of 300 µm in the axial plane (CBCT of TMJ in sagittal view). B. Schematic diagram showing slicing (S) in the axial plane. TMJ: temporomandibular joint; CBCT: cone-beam computed tomography Image B has been created by the author.

A systematic approach was employed to calculate the volume of the TMJ space. In the axial plane, a line was drawn through the deepest point of the glenoid fossa parallel to the orbital (Or) to the porion (Po) B’ line, which served as the axial plane reference. Another line, AA, which is termed the eminence plane, is defined as passing through the lowest point of the articular eminence. The space between these two parallel lines was divided into slices of 300 µm thickness (Figure 1).

**Figure 2:**
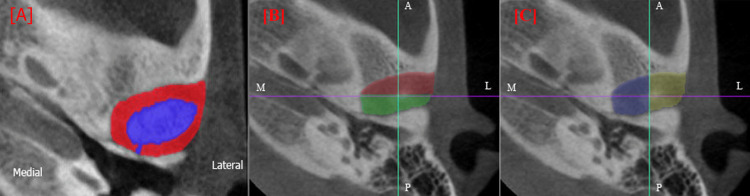
Three-dimensional volumetric measurement of the TMJ space from the axial plane in CBCT (A) Volume of the glenoid fossa (combined red and blue) and the TMJ space (only red area); (B) Division of the TMJ space in the anteroposterior direction; (C) Division of the TMJ space in the mediolateral direction A: anterior; P: posterior; M: medial; L: lateral; TMJ: temporomandibular joint; CBCT: cone-beam computed tomography

The area within the boundary of the glenoid fossa of each slice was calculated using ImageJ software. The volume of each slice was determined using the following formula: volume of each slice = slice thickness × area. The total volume of the space was calculated as the sum of the volumes of all slices between the two parallel lines: volume of space = ∑n (volume of the slice). The total volume of the glenoid fossa was computed by summing the areas of the two regions (the red and blue areas in Figure 2A) multiplied by the slice thickness.

The TMJ space volume (red area) was then calculated by subtracting the condyle volume (blue area) from the glenoid fossa volume, as follows: Volume of TMJ space = [area of the glenoid fossa (Red + Blue) − condyle area (blue)] × slice thickness. To further analyze the TMJ space, it was divided into specific regions. The anterior and posterior joint spaces were delineated using a mediolateral (ML) line parallel to the coronal plane and passing through the condylar center. The anterior space is represented in red, and the posterior space is represented in green in Figure 2B. Similarly, the medial and lateral joint spaces were defined using an anteroposterior (AP) line parallel to the sagittal plane, which also passed through the condylar center. The medial space is marked in blue, whereas the lateral space is indicated in yellow (Figure 2C). All measurements were performed by the same trained examiner (BP), who was blinded to the group allocation, and the measurements were repeated at two-week intervals for 10 randomly selected CBCT records. The intra-class correlation coefficient value of 0.92 showed high reliability and reproducibility. The shape of the mandibular condyle was also assessed as classified by Yale et al. [10] as oval, flat, round, and bird beak.

Statistical analysis

The statistician (JK) was blinded to group allocation and has been provided with coded data. The collected measurements were systematically entered into a Microsoft Excel spreadsheet (Microsoft Corp., Redmond, WA) and subsequently analyzed using IBM SPSS Statistics software, version 23 (IBM Corp., Armonk, NY, USA). Descriptive statistics, including frequency distributions, means, and standard deviations (SD), were used to summarize the data. The study groups, consisting of Class I and Class II malocclusion patients, were compared using a two-way analysis of independent t-tests. Statistical significance was determined at a threshold of p < 0.05. Additionally, regression analysis was conducted to identify the variable that most strongly influenced the volume of the TMJ space.

## Results

Among the 40 CBCT records of the TMJ included in the study, 20 cases (50%) were classified as Class I, and 20 cases (50%) as Class II. The sample comprised 21 males (52.5%) and 19 females (47.5%). The average age of participants in skeletal Class I patients was 27.30±2.62 years, whereas skeletal Class II patients had an average age of 27.70±2.77 years, demonstrating no statistically significant difference. Skeletal Class I patients showed statistically significantly greater TMJ space volume of 1621.45±138.06 mm³, compared to 1483.55±138.29 mm³ in skeletal Class II patients (p=0.003). Likewise, the volume of the anterior and medial TMJ space was significantly larger in Class I (878.35 ± 80.61 mm³ and 855.40 ± 76.63 mm³, respectively), in contrast to Class II (614.65 ± 65.81 mm³ and 532.85 ± 73.16 mm³, respectively). This showed that the condyle was placed anteriorly and medially in skeletal Class II patients. Cephalometric parameters, such as SNA, SNB, and ANB angles, revealed no significant difference between the groups (p > 0.05), as shown in Table 2.

**Table 2:** Comparison of study groups with independent T test p<0.05: significant (S); p>0.05: non-significant (NS); CI: confidence Interval; data are presented in form of n (%); mean ± standard deviation (SD)

		N	95% CI for mean	Mean ± SD	t value	p-value
Age (in years)	Class 1	20	26.07 - 28.53	27.30 ± 2.62	0.887	0.381(NS)
	Class 2	20	26.40 – 29.00	27.70 ± 2.77		
Total volume (in mm^3^)	Class 1	20	1556.84 - 1686.06	1621.45 ± 138.06	3.156	0.003(S)
	Class 2	20	1418.83 - 1548.27	1483.55 ± 138.29		
Anterior space volume (in mm^3^)	Class 1	20	840.62 - 916.08	878.35 ± 80.61	11.332	0.001(S)
	Class 2	20	583.85 - 645.45	614.65 ± 65.81		
Posterior space volume (in mm^3^)	Class 1	20	708.05 - 778.15	743.10 ± 74.90	-5.08	0.001(S)
	Class 2	20	830.73 - 907.07	868.90 ± 81.57		
Medial space volume (in mm^3^)	Class 1	20	819.53 - 891.27	855.40 ± 76.63	13.615	0.001(S)
	Class 2	20	498.61 - 567.09	532.85 ± 73.16		
Lateral space volume (in mm^3^)	Class 1	20	730.28 - 801.82	766.05 ± 76.43	-7.491	0.001(S)
	Class 2	20	916.73 - 995.07	955.90 ± 83.70		
SNA angle (in degree)	Class 1	20	82.69 - 83.84	83.27 ± 1.22	0.411	0.683(NS)
	Class 2	20	82.56 - 83.73	83.15 ± 1.24		
SNB angle (in degree)	Class 1	20	79.29 - 80.43	79.86 ± 1.22	1.179	0.246(NS)
	Class 2	20	74.21 - 75.77	74.99 ± 1.66		
ANB angle (in degree)	Class 1	20	3.16 - 3.65	3.41 ± 0.53	-1.093	0.293(NS)
	Class 2	20	7.53 - 8.78	8.15 ± 1.35		

The overall volume of the space demonstrated a significant positive correlation with the morphology of the condyle, SNA, and SNB angle. This showed that an oval condylar shape was associated with an increased total space volume. Age and sex did not show any significant correlation with TMJ volume. The ANB angle showed a significant negative correlation with total, anterior, and medial space volumes. The anterior and medial space volumes showed a significant positive correlation with the SNB angle and a significant negative correlation with posterior and lateral space volumes. This showed that retrognathic mandibles correlated with anteriorly and medially placed condyles (Table 3).

**Table 3:** Correlation analysis between independent and dependent factors *Pearson correlation; **Eta correlation; ***Point biserial correlation; p<0.05: significant (S)

Variables	Total space volume in mm^3 ^(r-value)	p-value	Anterior space volume in mm^3 ^(r-value)	p-value	Posterior space volume in mm^3 ^(r-value)	p-value	Medial space volume in mm^3 ^(r-value)	p-value	Lateral space volume in mm^3 ^(r-value)	p-value
Age *	0.08	0.629	-0.05	0.77	0.19	0.233	-0.04	0.815	0.19	0.245
SNA angle*	0.03	0.038 (S)	0.17	0.297	0.25	0.123	0.15	0.358	0.18	0.275
SNB angle*	0.47	0.002 (S)	0.77	0.001 (S)	-0.44	0.004 (S)	0.8	0.001 (S)	-0.58	0.001 (S)
ANB angle*	-0.36	0.023 (S)	-0.76	0.001 (S)	0.6	0.001 (S)	-0.81	0.001 (S)	0.72	0.001 (S)
Condyle shape**	0.29	0.003 (S)	0.06	0.026 (S)	0.36	0.323	0.06	0.613	0.28	0.226
Gender***	-0.14	0.396	-0.18	0.258	0.07	0.68	-0.15	0.36	0.02	0.882

Multivariate linear regression analysis indicated significant correlations between malocclusion (Class II) and anterior space volume (β=−354.77, p=0.001), medial space volume (β=−396.81, p=0.001), and lateral space volume (β=198.45, p=0.006). Additionally, malocclusion was significantly correlated with the posterior space volume (β=125.38, p=0.06). Other factors, such as age, gender, SNA angle, SNB angle, ANB angle, and condyle morphology, did not reveal statistically significant correlations across space volume parameters. These results imply that malocclusion is a crucial determinant affecting the TMJ space volume (Table 4).

**Table 4:** Multivariate regression analysis showing the relationship between demographic, skeletal, and condylar parameters with TMJ space volumes p<0.05: significant (S); TMJ: temporomandibular joint

Parameters	Anterior space volume in mm^3^		Posterior space volume in mm^3^		Medial space volume in mm^3^		Lateral space volume in mm^3^	
	Coefficients	p-value	Coefficients	p-value	Coefficients	p-value	Coefficients	p-value
Age in years	-0.34	0.944	2.34	0.64	1.27	0.796	2.2	0.668
Gender (female)	-22.24	0.375	-1.04	0.97	-10.43	0.68	-16.35	0.535
Malocclusion (skeletal Class II)	-354.77	0.001(S)	125.38	0.06	-396.81	0.001(S)	198.45	0.006(S)
SNA angle in degrees	12.03	0.132	10.35	0.2	9.5	0.237	9.99	0.232
SNB angle in degrees	-3.61	0.55	5.2	0.4	-2.64	0.667	5.5	0.39
ANB angle in degrees	15.64	0.069	5.16	0.55	12.14	0.159	4.48	0.612
Condyle shape (flat)	-21.44	0.528	-63.74	0.07	-34.4	0.321	-55.86	0.125
Condyle shape (round)	30.19	0.354	-14.84	0.66	42.46	0.203	-31.05	0.367
Condyle shape (bird beak)	-7.42	0.869	-48.77	0.3	-4.34	0.924	-56.04	0.244

The graph depicts the distribution of mandibular condyle shapes, analyzed by gender and malocclusion classification. Among male participants, the oval condyle shape was the most frequently observed, occurring in 12 cases (30.00%), followed by the flat shape in four cases (10.00%), the round shape in three cases (7.50%), and the bird beak shape in two cases (5.00%). In female participants, the oval shape was identified in nine cases (22.50%), the flat shape in three cases (7.50%), the round shape in five cases (12.50%), and the bird-beak shape in two cases (5.00%).

**Figure 3:**
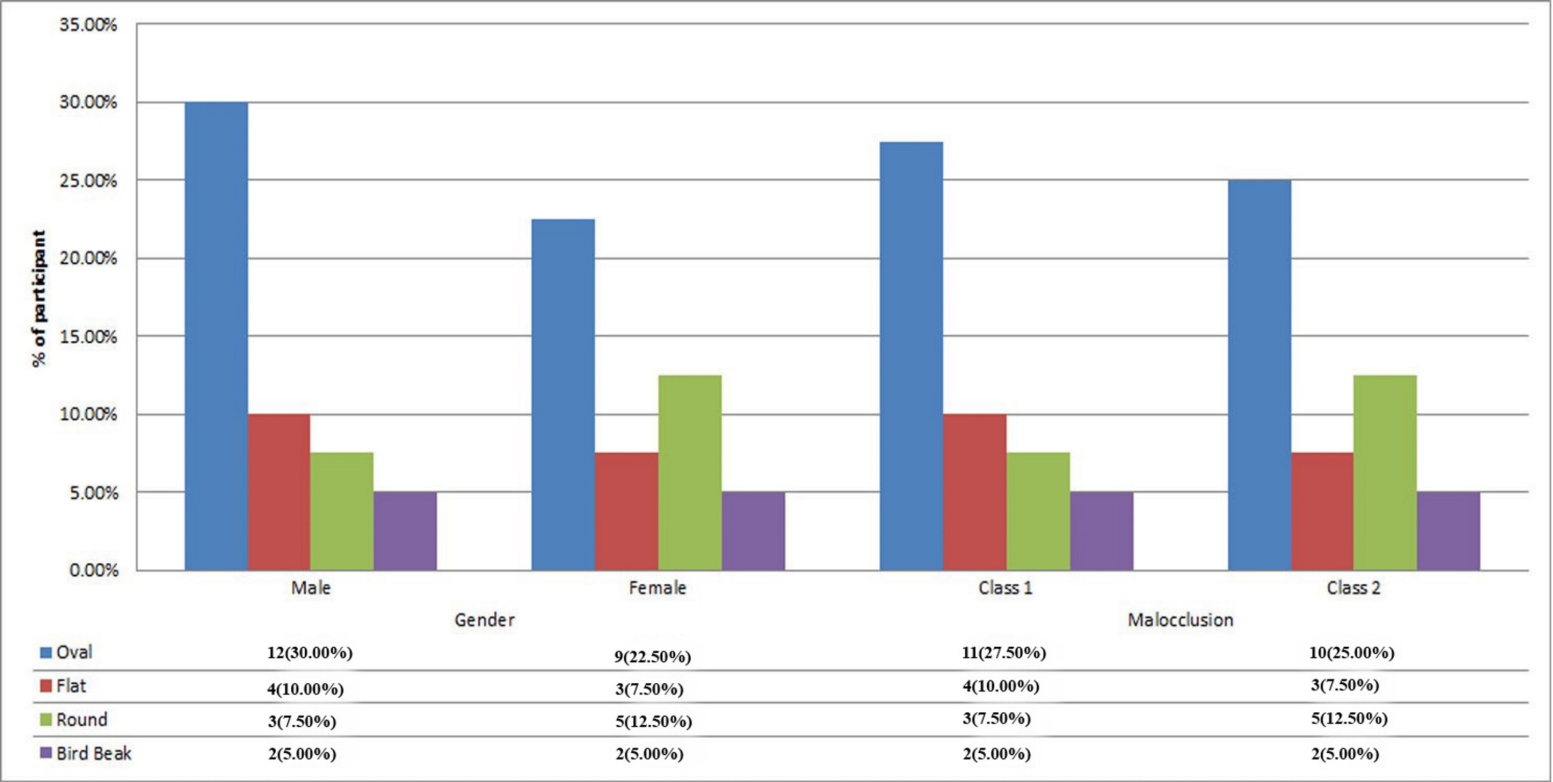
Frequency distribution of participants into study groups considering the shape of mandible

In individuals with malocclusion, the oval shape predominated, appearing in 11 cases (27.50%) among Class I and 10 cases (25.00%) among Class II malocclusion. This was followed by the flat shape, observed in four cases (10.00%) in Class I and three cases (7.50%) in Class II. The round shape was noted in three cases (7.50%) in Class I and five cases (12.50%) in Class II. The bird-beak shape was the least common, with two cases (5.00%) in both malocclusion classes (Figure 3).

## Discussion

The findings of our study provide valuable insights into the variations in TMJ space volume and mandibular condyle morphology among skeletal Class I and Class II malocclusion patients, which will help expand our understanding of how malocclusion influences TMJ anatomy and spatial relationships, emphasizing its clinical relevance in orthodontic diagnosis and treatment planning.

Age and gender effects

The study found no statistically significant correlation between age, sex, and TMJ space volumes. These findings suggest that age-related degenerative changes or gender-based anatomical differences do not play a critical role in influencing TMJ space volume in this specific population. Liu et al. also found no statistically significant differences in condylar position with advancing age [11]. However, some previous studies have suggested that age and hormonal variations, such as estrogen levels in women, might affect TMJ morphology and function; these factors were not significant determinants in the present study [12]. This could be attributed to the relatively narrow age range and balanced sex distribution of the study cohort, which likely minimized such effects.

Space volume differences of the TMJ

One of the most significant deductions derived from this analysis was the variance in the TMJ space volumes between individuals classified as skeletal Class I and Class II. Subjects identified as skeletal Class I demonstrated a markedly greater total TMJ space volume when juxtaposed with those categorized as skeletal Class II. Importantly, the anterior and medial TMJ spaces were notably more pronounced in Class I subjects, suggesting that Class II individuals frequently exhibit anterior and medial condylar positioning. This finding is supported by previous studies [1,13]. These results align with earlier research that recognized a decrease in the anterior TMJ space among patients experiencing Class II malocclusion, which may predispose these individuals to TMJ dysfunction or increased joint stress in the anterior and medial compartments [14].

Condyle morphology and TMJ space

The present study elucidated a significant correlation between the anatomical structure of the condyle and TMJ space. Specifically, rounded condyle morphology was associated with an increased overall volume of the TMJ space. This finding suggests that the configuration of the condyle may influence the spatial organization of the TMJ, with oval-shaped condyles demonstrating enhanced alignment to support the optimal joint space. Conversely, other configurations, such as flat or beak-like condyles, may reflect anatomical modifications in response to altered loading conditions or growth patterns evident in individuals with malocclusion [15]. The oval type was the most prevalent condylar shape in our sample, as in a previous study [16].

Malocclusion and TMJ space volumes

Multivariate regression analysis revealed malocclusion as a significant factor influencing TMJ space volumes. The modified positioning of the condyle is presumably indicative of the retrognathic growth pattern commonly associated with Class II malocclusion, characterized by medial and anterior displacement of the mandibular condyle within the glenoid fossa [17]. From a clinical standpoint, these findings underscore the necessity for meticulous evaluation of TMJ anatomy in individuals presenting with skeletal Class II malocclusion. A diminished TMJ space may render these subjects susceptible to biomechanical discrepancies, thereby potentially elevating the likelihood of joint pathologies such as internal derangements, disc displacement, or osteoarthritis of the TMJ. Furthermore, the altered spatial dynamics identified in Class II patients could bear significant ramifications for orthodontic treatment strategies, particularly in the management of mandibular retrognathism or when considering interventions such as functional appliances or orthognathic surgical procedures.

Clinical implications

The results of this investigation have numerous clinical ramifications concerning the diagnosis, treatment, and management of patients with skeletal malocclusions. Initially, the diminished volumes of the TMJ space identified in Class II subjects underscored the necessity for comprehensive TMJ assessments in these patients, particularly when devising treatment strategies that modify mandibular positioning. For example, functional appliances are designed to rectify mandibular retrognathism and may potentially enhance the dynamics of the TMJ space, thereby mitigating the likelihood of joint dysfunction.

The discrepancies in condylar morphology identified in this investigation indicate that structural alterations may manifest as adaptive mechanisms that modify skeletal interactions. Comprehending these adaptations is imperative for forecasting therapeutic outcomes and mitigating potential complications, especially in individuals with significant skeletal anomalies or chronic malocclusions.

Limitations and future directions

The relatively small sample size and narrow age demographics may restrict the generalizability of our findings to broader populations. Furthermore, the cross-sectional design of the study inhibits the assessment of longitudinal changes in the TMJ structure and condylar morphology, which would enhance a more dynamic understanding of these relationships. Additionally, our analysis excluded subjects with skeletal Class III traits and diverse growth patterns.

Subsequent research should concentrate on more extensive and heterogeneous populations while integrating longitudinal frameworks to assess temporal changes in TMJ spatial dimensions and morphology. Furthermore, investigations examining the functional implications of modified TMJ anatomy, including joint loading dynamics and susceptibility to TMJ disorders, would yield a significant understanding of the clinical importance of these observations.

## Conclusions

In conclusion, this study underscores marked disparities in the volumes of the TMJ space and morphology of the condyle between patients classified as skeletal Class I and Class II, with malocclusion identified as a pivotal factor influencing TMJ anatomy. The oval shape of the condyle was the most frequently observed morphology in the present study. Notably, the anterior and medial volumes of the TMJ space exhibited a significant increase in skeletal Class I individuals. Furthermore, there was no substantial correlation between TMJ space volume, age, and sex.

## References

[1] Rivero-Millán P, Barrera-Mora JM, Espinar-Escalona E, González-Del Pino CA, Martín-Salvador D, Llamas-Carreras JM (2021). Comparison of condylar position in normal occlusion, class II division 1, class II division 2 and class III malocclusions using CBCT imaging. J Clin Exp Dent.

[2] Dhabale GS, Bhowate RR (2022). Cone-beam computed tomography for temporomandibular joint imaging. Cureus.

[3] Arieta-Miranda JM, Silva-Valencia M, Flores-Mir C, Paredes-Sampen NA, Arriola-Guillen LE (2013). Spatial analysis of condyle position according to sagittal skeletal relationship, assessed by cone beam computed tomography. Prog Orthod.

[4] Sharma A, Pai V, Hegde M, Rajaram S (2022). Three-dimensional evaluation of condylar position in skeletal Class I and Class II malocclusions along with vertical facial morphology. APOS Trends Orthod.

[5] Zhang ZL, Cheng JG, Li G, Zhang JZ, Zhang ZY, Ma XC (2012). Measurement accuracy of temporomandibular joint space in Promax 3-dimensional cone-beam computerized tomography images. Oral Surg Oral Med Oral Pathol Oral Radiol.

[6] García-Sanz V, Bellot-Arcís C, Hernández V, Serrano-Sánchez P, Guarinos J, Paredes-Gallardo V (2017). Accuracy and reliability of cone-beam computed tomography for linear and volumetric mandibular condyle measurements. A human cadaver study. Sci Rep.

[7] Riesmeijer AM, Prahl-Andersen B, Mascarenhas AK, Joo BH, Vig KW (2004). A comparison of craniofacial Class I and Class II growth patterns. Am J Orthod Dentofacial Orthop.

[8] Kim JY, Yong HS, Kim TY, Kim JY, Jeon KJ, Huh JK (2024). Volumetric changes in temporomandibular joint space following trans-oral vertical ramus osteotomy in patients with mandibular prognathism: a one-year follow-up study. Sci Rep.

[9] Kim JY, Kim BJ, Park KH, Huh JK (2016). Comparison of volume and position of the temporomandibular joint structures in patients with mandibular asymmetry. Oral Surg Oral Med Oral Pathol Oral Radiol.

[10] Yale SH, Allison BD, Hauptfuehrer JD (1966). An epidemiological assessment of mandibular condyle morphology. Oral Surg Oral Med Oral Pathol.

[11] Liu Q, Wei X, Guan J, Wang R, Zou D, Yu L (2018). Assessment of condylar morphology and position using MSCT in an Asian population. Clin Oral Investig.

[12] Smartt JM Jr, Low DW, Bartlett SP (2005). The pediatric mandible: I. A primer on growth and development. Plast Reconstr Surg.

[13] Krisjane Z, Urtane I, Krumina G, Zepa K (2009). Three-dimensional evaluation of TMJ parameters in class II and class III patients. Stomatologija.

[14] Rathi S, Gilani R, Kamble R, Bhandwalkar S (2022). Temporomandibular joint disorder and airway in class II malocclusion: a review. Cureus.

[15] Al-Rawi NH, Uthman AT, Sodeify SM (2017). Spatial analysis of mandibular condyles in patients with temporomandibular disorders and normal controls using cone beam computed tomography. Eur J Dent.

[16] Dahal S, Atreya A, Prasad Gupta S, Natarajan S (2022). Oval type of human mandibular condyle in panoramic radiographs of a tertiary care centre: a descriptive cross-sectional study. JNMA J Nepal Med Assoc.

[17] Al-Hadad SA, ALyafrusee ES, Abdulqader AA, Al-Gumaei WS, Al-Mohana RA, Ren L (2022). Comprehensive three-dimensional positional and morphological assessment of the temporomandibular joint in skeletal class II patients with mandibular retrognathism in different vertical skeletal patterns. BMC Oral Health.

